# Impact of Parameter Variation in Fabrication of Nanostructure by Atomic Force Microscopy Nanolithography

**DOI:** 10.1371/journal.pone.0065409

**Published:** 2013-06-11

**Authors:** Arash Dehzangi, Farhad Larki, Sabar D. Hutagalung, Mahmood Goodarz Naseri, Burhanuddin Y. Majlis, Manizheh Navasery, Norihan Abdul Hamid, Mimiwaty Mohd Noor

**Affiliations:** 1 Institute of Microengineering and Nanoelectronics, Universiti Kebangsaan Malaysia, Bangi, Selangor, Malaysia; 2 School of Materials and Mineral Resources Engineering, Universiti Sains Malaysia, NibongTebal, Penang, Malaysia; 3 Department of Physics, Faculty of science, Universiti Putra Malaysia, Serdang, Selangor, Malaysia; 4 Department of Physics, Faculty of Science, Malayer University, Malayer, Hamedan, Iran; Harbin Institute of Technology, China

## Abstract

In this letter, we investigate the fabrication of Silicon nanostructure patterned on lightly doped (10^15^ cm^−3^) p-type silicon-on-insulator by atomic force microscope nanolithography technique. The local anodic oxidation followed by two wet etching steps, potassium hydroxide etching for silicon removal and hydrofluoric etching for oxide removal, are implemented to reach the structures. The impact of contributing parameters in oxidation such as tip materials, applying voltage on the tip, relative humidity and exposure time are studied. The effect of the etchant concentration (10% to 30% wt) of potassium hydroxide and its mixture with isopropyl alcohol (10%vol. IPA ) at different temperatures on silicon surface are expressed. For different KOH concentrations, the effect of etching with the IPA admixture and the effect of the immersing time in the etching process on the structure are investigated. The etching processes are accurately optimized by 30%wt. KOH +10%vol. IPA in appropriate time, temperature, and humidity.

## Introduction

The conventional techniques for semiconductor fabrication are designed in various lithographical methods based on the top-down approach. These techniques are highly developed and optimized. However, their high operating cost, multiple-step processes, and poor accessibility for nanofabrication regularly limit the applicability of these techniques. Unconventional methods, on the other hand, can provide simpler and cheaper courses to the fabrication of nanostructures. Recently, novel methods such as nano-imprint lithography (NIL) [1], [2], soft lithography [3], and atomic force microscopy (AFM) nanolithography [4], [5] have been emerged as flexible alternatives for nanoscale patterning and fabrication. These novel methods have the potential to be as future low-cost techniques in nanoscale pattern formation and replication. Among these techniques, AFM nanolithography techniques can be capable of fabricating or characterizing microarrays [6], nanoscale structures, sensors or devices.

Generally, AFM based techniques can be categorized into three main groups in terms of their operational principles as force-assisted, bias-assisted [7] and thermal-assisted [8] AFM nanolithography. Tip induced local anodic oxidation (LAO), as a bias-assisted method, is one of the widely investigated processes for AFM nanolithography. Local oxidation of silicon surfaces by using AFM lithography techniques in the air, was observed in 1994 by Snow and Campbell [9] for the first time, when they realized that it had a numerous advantage over the previous scanning tunneling microscope (STM) based method [10]. Proper choice and preparation of a sample are of great importance in LAO. Although, the first experiments started on a Si (111) surface, a hydrogen passivated p-type Si (100) was subjected to many works for LAO [11], [12]. Ionica et al. [13] have remarkably reported the electrical characteristics of the devices made by AFM nanolithography. In addition, some recent works were performed to improve the method of AFM nanolithography [14], [15].

The widely used wet etching is still an applicable technique in the fabrication of Si nanostructures. Tetramethyl-amino-hydroxide (TMAH) and potassium hydroxide (KOH) saturated with Isopropyl alcohol (IPA) are two well-known etchants, applicable for etching nanostructures fabricated by LAO technique. We reported the electrical property and fabrication of the p-type side gate nanowire junctionless transistor (JLT) by AFM – LAO nanolithography, on p-type (100) silicon on insulator (SOI), using KOH+IPA wet etching [16]. The numerical performance of the devices, regarding to the particular method of fabrication (AFM nanolithography), were also investigated for the single gate [17] and double gate structure [18]. The structure and particularly nanowire are defined by well precise crystalline plains where the etching is supposed to be stopped due to the anisotropic etching. The regularity and controllability of the cross section of the nanowire (trapezoidal cross section) after etching can be an important advantage for the fabrication of ultra-small and long nanostructures [19].

In this work, we present an elaborate investigation of experimental parameter variation impact on the fabrication of nanodevices by AFM nanolithography. To complete the previous works as a guide for future experiments, comprehensive information and a precise view of every step of bias-assisted AFM nanolithography are provided in details. Meanwhile, we will review some of the previous works to support or make comparisons. The important contributing parameters in the fabrication process, from preparation of the SOI wafer to the final extraction of the structure are presented as well. In this matter, the effect of tip materials, applying voltage on the tip, relative humidity (RH%) and the exposure time in the LAO process, as well as the effect of KOH concentration and immersing time on the SOI surface and the quality of etched structure, are investigated.

## Methodology

A nanostructure was fabricated via AFM nanolithography by scanning probe microscope machine (SPI3800N/4000, SII Nanotechnology Inc., Chiba-shi, Chiba, Japan). Local anodic oxidation was carried out on a lightly doped (10^15^ cm^−3^) p-type B-doped (100) SOI wafer (prepared by Unibond™ (Unibond International Ltd., Uxbridge, Middlesex, UK)), with a top silicon of 100 nm and a 145 nm buried oxide (BOX) thickness with a resistivity of 13.5–22.5 Ω cm (Soitech) [20]. The LAO procedure was performed in the contact mode, employing AFM conductive-coated tips with the force constant of 0.2 N m^−1^ and the resonance frequency of 13 kHz. The important parameters for the scan console during the AFM scanning in different scan area are illustrated in Table 1.

**Table 1:** The outline of the scan parameters for the AFM scanning.

Scan area (nm)	200	500	2000	1000
**Scan speed (Hz)**	6	4	2	1
**I Gain**	0.15	0.20	0.30	0.40
**P Gain**	0.05	0.10	0.15	0.20

Room humidity (RH) was controllable from 50% to 80% with an accuracy of 1%. After the oxidation procedure, two steps of the wet etching process were applied to extract the nanostructure. In this study, the SiO_2_ formed by LAO is considered as a mask on the top of the SOI surface. The unoxidized Si is the undesired area, which is supposed to be removed by KOH solution (KOH pellets of 56.11 g/mol supplied by System) as the etchant. The BOX layer was used as the etch-stop in the wet - etching process. All KOH etching experiments were carried out in a closed glass beaker with a constant temperature bath.

The oxide removal is the second step in the etching process and the last step in our fabrication method. The etchant is hydrofluoric acid (HF, 49% concentration supplied by JT Baker) in aqueous solution with the well-known ability to dissolve SiO_2_. During HF etching, the oxide mask and the native oxide were removed.

**Figure 1:**
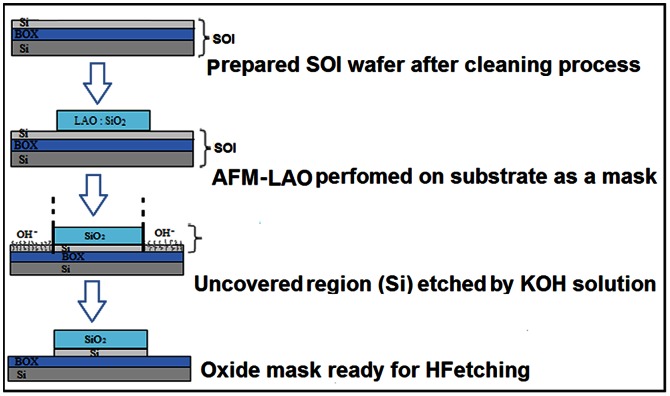
Schematic steps of Fabrication by AFM-LAO nanolithography.

The whole process of fabrication can be divided into three separate and major steps, which are sample preparation, oxide growth mechanism on the desired area (mask) and etching processes (schematically illustrated in Fig. 1). Each step has important contributing parameters and a significant impact on the fabrication of the final structure, which will be addressed in the next section. The shape of the device consists of square pads as the contacts (source, drain and gate) and a nanowire between the square pads as the channel [16]. The morphology of the etched surfaces was also observed by using scanning electron microscopes (JEOL JSM-6360).

## Results and Discussion

### Cleaning and Sample Preparation

The sample preparation is an important part in nanofabrication. A silicon surface in air ambient is covered by a variety of contaminations, such as organic and inorganic contaminations as well as native oxide. In order to obtain a nanometer flat oxide-resistant passivated surface for LAO, it is necessary to provide contaminant elimination, native oxide removal, and silicon dangling bonds saturation by hydrogen atoms [11]. Werner Kern developed one of the standard cleaning procedures in industry in 1965, while he was working at the Radio Corporation of America (RCA). It is known as the RCA method [21]. The RCA cleaning process is implemented in sample preparation as a reliable method. Prior to RCA, the samples are exposed to ultrasonic energy in an ultrasonic bath. The RCA method was modified and optimized to extract the best possible output, especially for the SOI sample with a thin layer of Si. The modified RCA applied to the samples is summarized in Table 2 in three separate steps. During the optimization of the cleaning process, major parameters were the ratio of chemical concentration with deionized water (DIW), temperature, and time.

**Table 2:** Steps of the modified RCA method used in the preparation process.

Step1	Step2	Step 3
Solution of DIW: H_2_O_2_: NH_4_OH, (5∶1:1) was preparedin the glass beaker and heated to 77 to 80°C	Solution of 6∶1:1 DIW:H_2_O_2_:HCl, was prepared inthe glass beaker and heated up to 77 to 80°C	Solution of HF: DIW with a ratio of 1∶100 was prepared in the polypropylene beaker
The wafers were soaked for 12 minutes and the temperature was maintained not more than 80°C	The wafers were soaked for 12 minutes at temperature not more than 80°C	The wafers were soaked in the solution for 20 −30 seconds, until the wafer became hydrophobic
The wafer was rinsed with DIW for 1 minute	The wafer was rinsed in DIW for 1 minute	The wafer was rinsed in DIW for 1 minute and immersed for 2–3 minutes

In the first step of the cleaning process, the ammonia/peroxide mixture (APM) oxidizes the silicon surface using hydrogen peroxide (H_2_O_2_), and simultaneously removes the silicon oxide by means of ammonium Hydroxide (NH_4_OH, concentration of 25%wt) at alkaline pH [22]. In the second step of cleaning to remove ionic contamination by using hydrochloric/peroxide mixture (HPM), metal ions and metal hydroxides will be solubilized. Hydrochloric acid (HCl) generates a metal cation in solution that forms an ionic bond with the chloride anion in order to rinse all metal tracks off the silicon surface [23].

**Figure 2:**
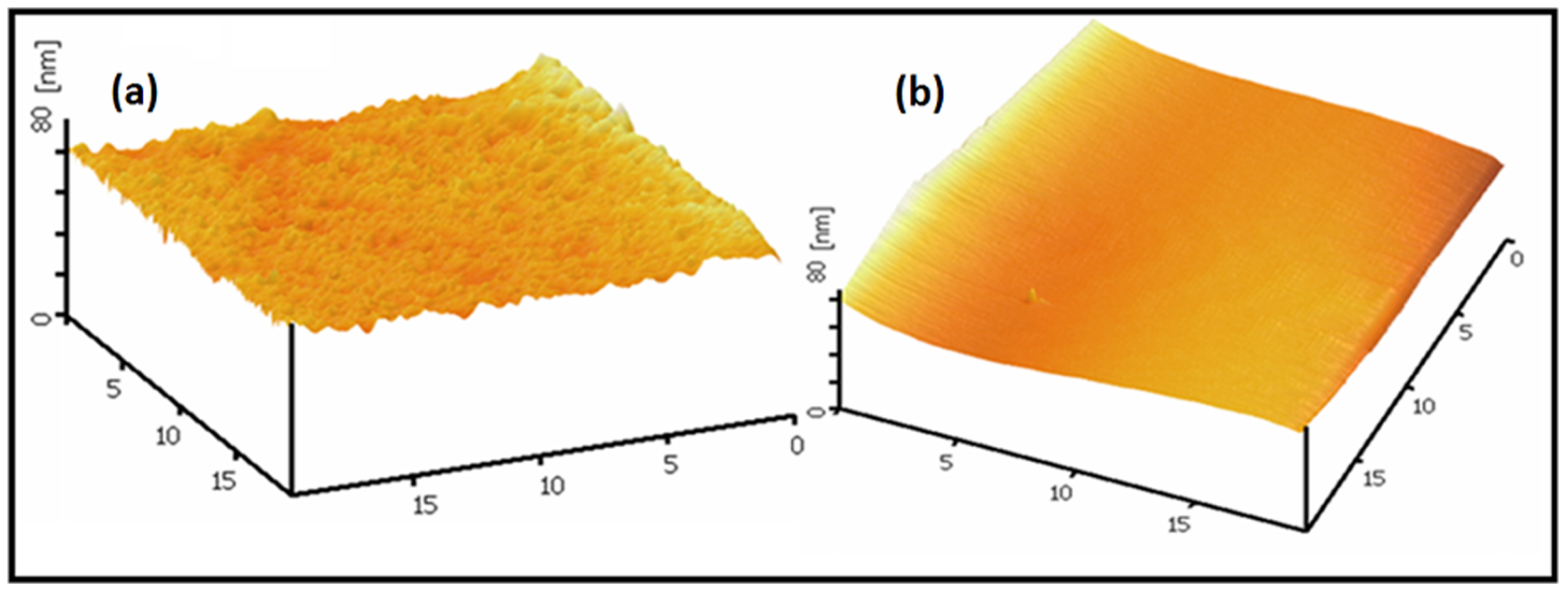
AFM images showing the effect of improper cleaning parameters on the SOI surface (a) long time interval (25 min), and (b) high temperature (>100 °**C).**

In this step, temperature and time interval control play a crucial role in the nanometer flatness of the silicon surface. Fig. 2 shows the effect of improper temperature and time interval on Si surface. For a solution with longer time interval (25 min), the surface roughness increases (Fig. 2a) and for a solution with a higher temperature (>100°C), the surface flatness degrades (Fig. 2.b). Any change in temperature more than 3°C during the cleaning process (when the temperature exceeds 80°C) would give rise to inequalities or even twists on the sample surface.

**Figure 3:**
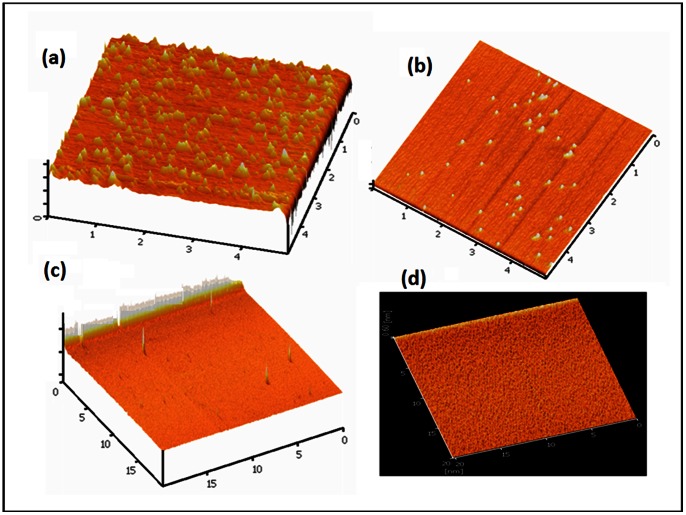
The effect of HF concentration on native oxide removal during the sample preparation.

To remove the native oxide and saturate the silicon dangling bonds, concerning (100) silicon surface, an aqueous HF (concentration of 49%) solution was employed. Fig. 3(a–d) demonstrate the impact of HF concentration variation, from 0.25% to 1.0% in DIW (in the steps of 0.25%) at the temperature of 242°C and humidity of 672%.

**Figure 4:**
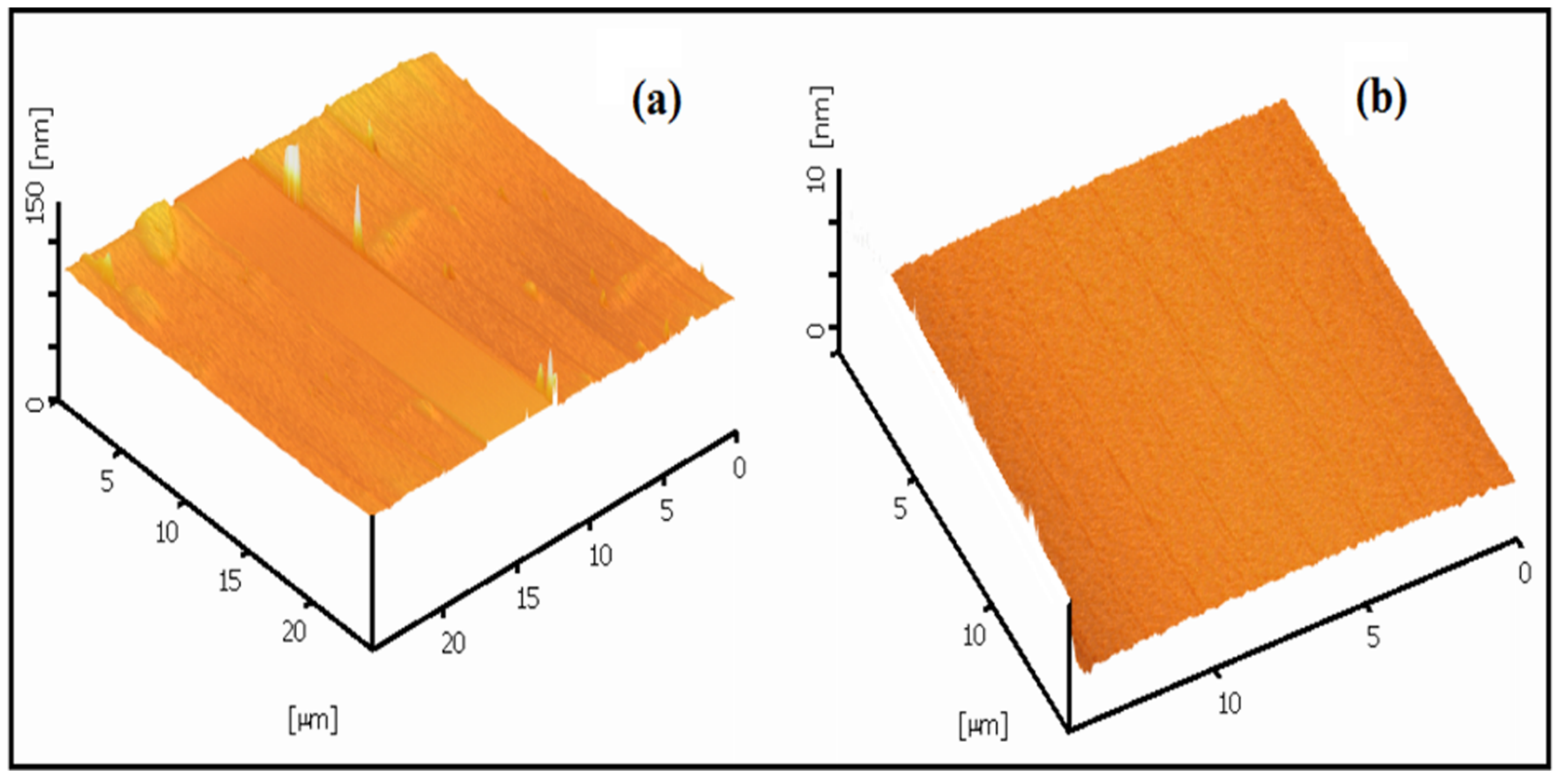
AFM images of the SOI substrate (a) before and b) after optimized RCA.

It was also found that the type and brand of SOI wafer can make some differences in the details of each step of the cleaning process. Fig. 4 shows the AFM images of the SOI arbitrary sample before and after the optimized cleaning process.

### LAO- AFM

**Figure 5:**
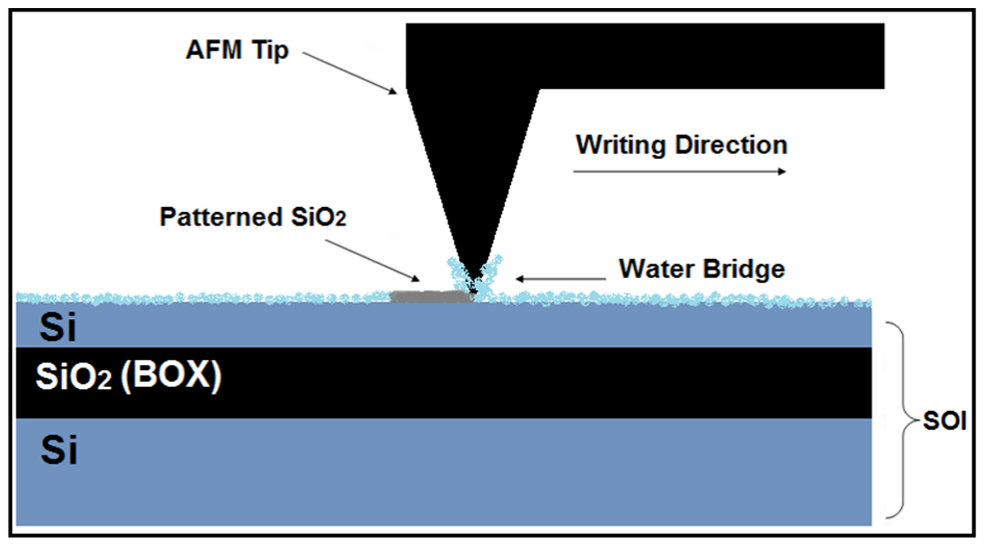
Schematic of AFM – LAO process on SOI substrate.

After the cleaning process, Si-H bonds would be formed on the surface of the SOI. When the surface was de-passivated, the first layer of Si-Si bonds became polarized due to the high electron negativity of the OH**^−^**. During LAO, a biased AFM tip (−5 to −15 V) is operating under an ambient humidity to locally oxidize the surface of a desired sample. For few nanometers gap between the tip and the surface, AFM tip bias would generate a field of 10^8^ V/m to 10^10^ V/m. The available water meniscuses, as a water bridge in the gap, are dissociated by this negative tip bias. Fig. 5 schematically shows the LAO-AFM process.

The oxide growth mechanism has been elaborately described in the literature [24], [25]. Complete understanding or quantifying process is complicated due to the specific spatial details of the tip/surface contact profile, as well as the cantilever motion under the applied bias [26]. However, some explanations and models are proposed to explain the anodic oxidation mechanism. In Table 3, some of the basic proposed models for the local oxidation by the AFM nanolithography are summarized. All the research groups agreed that the oxidation mechanism and kinetics are closely related to the electric field, surface stress, water meniscus formation, and OH**^−^**diffusion [27], [28], [29], [30].

**Table 3:** Various AFM local anodic oxidation models.

Oxidation model	Description
**Cebrera-Mott model**	The thickness of the oxide is governed by a diffusion limited of the electric field [31], [64]
**Power- law model**	The AFM oxidation is only observed for a voltage which exceeds more than a doping dependent threshold, above which the oxidation kinetics follows a power law [32]
**Log kinetic model**	The height of the oxide is proportional to log(1/t) and the linear behavior between 1/h vs log v [36]
**Space charge model**	i) Varied space-charge dependence of oxidation is a function of substrate doping type/level.
	ii) Space- charge effects are consisted of the rapid decline of the high initial growth rate.
	iii) The Alberty- Miller scheme describe a direct pathway for reaction of oxyanions with silicon at the Si/SiO_2_ interface, and an indirect reaction pathway, mediated by the trapping charge defects located at the interface [44], [65].

Several models have been proposed for explaining the oxide growth on the surface of semiconductors such as Cabrera–Mott model [31], power-law model [32], direct log kinetic model [33] and space charge model [34]. Marchi et al. [35] reported a significant increase of the oxide growth by an ozone supply in ambient air which implies that the oxide growth process is limited both thermodynamically and kinetically.

The Stievenard model [36], which is modified from the Cabrera-Mott model, is the best match with our experimental results, regarding to the quantitative analysis of the measured oxide height. Based on this model, the ionic diffusion of OH**^−^** and O**^−^** takes place through the oxide layer, from the oxide surface towards the Si/SiO_2_ interface. The applied electric field enhances the diffusion mechanism, due to the Mott potential difference between the initial oxidized silicon and the adsorbed oxygen layer [36]. Under a particular condition, which is extendable into our case, the height of the oxide thickness varies linearly with the applied voltage.

### AFM tip

In the LAO process, in addition to the major factors like RH%, applying voltage, exposure time or preparation process, some minor factors exist, which are able to make a significant effect in details. The type of the AFM tip can be very important since the coated layer of a tip enhances the laser reflectivity of the cantilever. The tip should be specified for the contact mode (tapping mode and non-contact mode tips are nonfunctional in this study). It is worth mentioning that for patterning very small oxide layer on the top of the SOI surface, some remarkable work has been performed by using the tapping mode or non-contact mode [37]. We examined three types of the contact mode tips, as the gold coated (Budgetsensors Au coated ContGB-G), the Chromium/Platinum coated conductive probe (Budgetsensors Cr/PtcoatedCont-G) and the Alumina reflex coated (Budgetsensors ContAl), among which the last one was not applicable. Table 4 illustrates the optimized parameter comparison during the LAO process for two different AFM tips. Both tips had the force constant (k_c_) of 0.2 N m^−1^ and the resonance frequency of 13 KHz (tip radius <25 nm).

**Table 4:** LAO Parameter comparison for Au and Cr/Pt coated AFM tip.

Type of AFM - tip	Applied voltage	Writing speed	Scan speed	Force reference
**Conductive Au-coated**	8 V	0.6 µm/s	1.0 µm/s	−0.2 N
**Conductive Cr/Pt coated**	9V	0.5 µm/s	1.0 µm/s	−0.1 N

### Applying Voltage at AFM Tip and Exposure Time (Writing Speed)

**Figure 6:**
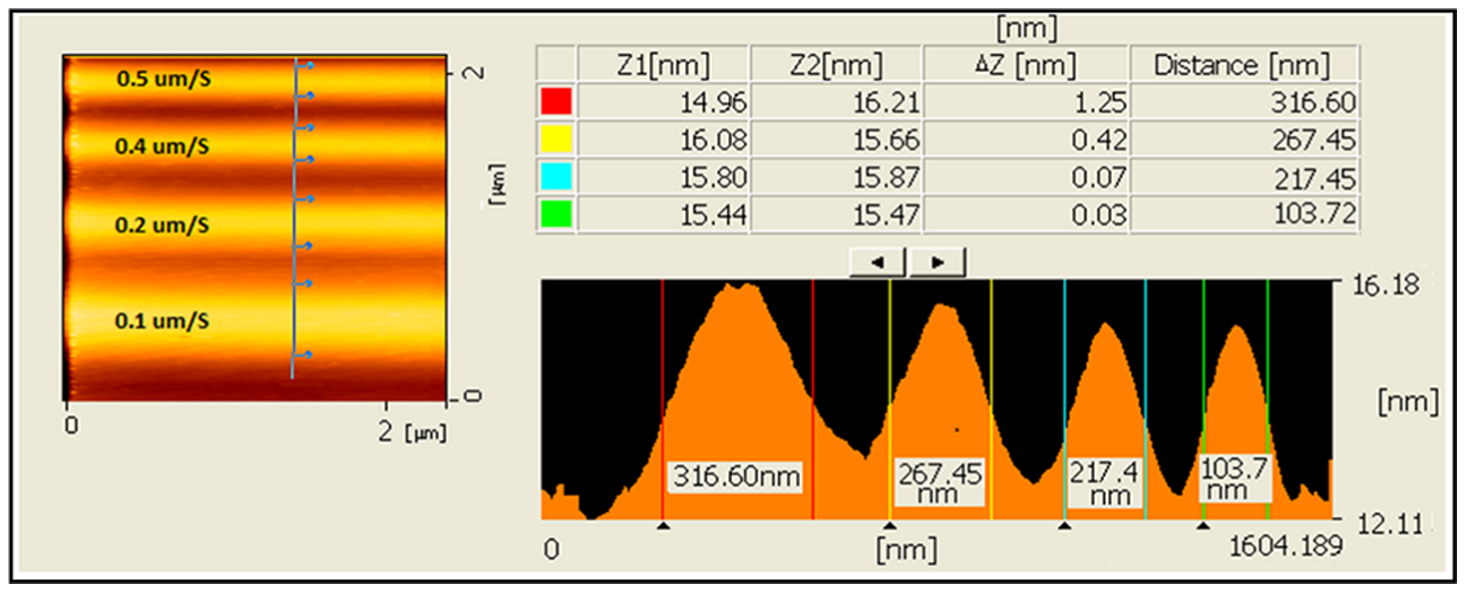
AFM profile image for width comparison of the oxide track for different writing speeds during LAO (Au coated tip at 8V applied voltage).

The writing speed and the AFM tip voltage are two major parameters to optimize the LAO process. A decrease was observed in the oxide height by increasing the writing speed. At the same humidity and temperature for Au coated tip, the width comparison of the oxidation tracks for different writing speeds is shown in Fig. 6c at RH 68% and 23°C with a constant applied voltage (8V).

Low writing speeds made wider oxidation tracks on the substrate, whereas faster writing speed might provide negligible track for constant applied voltage. It can be explained by the fact that as the writing speed increases, the numbers of diffusible ions into the Si/SiO_2_ interface decreases. For Cr/Pt coated tip with the writing speeds faster than 1.0 µm/s, the oxidation tracks were not recognizable. It was observed that in comparison with the Cr/Pt coated tip at the same applied voltage, the Au coated tip provided thicker oxide tracks for different writing speeds. Measured thicknesses at different writing speeds for two types of AFM tip are summarized in Table 5.

**Table 5:** Oxide thickness for different AFM tips (RH 68%, T = 23°C).

Writing speed	2.0 µm/s	1.5 µm/s	1.0 µm/s	0.8 µm/s	0.5 µm/s	0.3 µm/s
**Oxide thickness for Au-coated tip**	∼ 0	1.3 nm	2.4 nm	3.1 nm	3.2 nm	3.4 nm
**Oxide thickness for Cr/Pt coated tip**	∼ 0	1.0 nm	2.0 nm	2.5 nm	2.8 nm	3.0 nm

**Figure 7:**
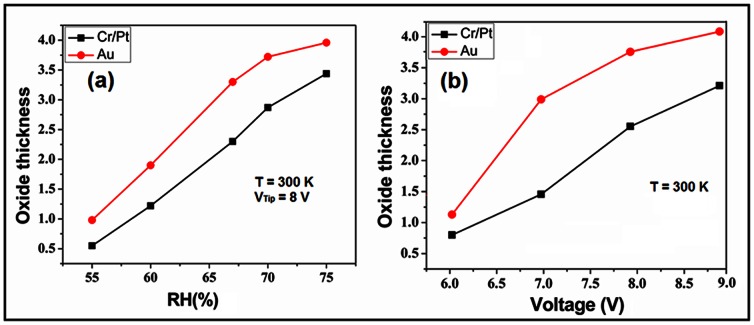
Relation of the oxide thickness (nm) with a) RH %, and b) applied voltage (68% of the RH%), for different AFM tips and the writing speed of 0.6 µm/s.

Regarding to the particular condition of the experiment, the best results were extracted by Cr/Pt coated conductive probe, since the quality of the oxide layer, in terms of uniformity or narrowness, was more applicable for the purpose of fabrication (especially after etching process). Au coated conductive probe provided a thicker oxide layer after oxidation, though with a lower oxide quality. A further comparison of the impact of different AFM tips on the oxide thickness will be addressed later (Fig. 7).

As mentioned before, the oxidation rate in AFM - LAO is directly proportional to the field strength and applying voltage on the tip. At a given writing speed, we observed that the oxide thickness is enlarged with the increase in the applied voltage. Interestingly, by increasing the applied voltage on the AFM tip, the width of the oxide tracks does not show any significant dependence which is in agreement with previous reports [38].

### Relative Humidity

Another parameter that plays a vigorous role in LAO process is RH%, which is due to the necessity of water molecules in an ambient air during the oxidation process. The oxidation rate is sensitive to humidity and the oxide thickness increases at high RH%. The impact of humidity on the oxidation procedure was previously reported [39]. For two different types of the AFM tip, at constant applied voltage (8V) and writing speed (0.6 µm/s), the relation between oxide thicknesses and RH% is shown in Fig. 7a (at room temperature). It can be seen that the oxide thickness increases as a linear function of RH%. Presence of the humidity around the electrodes influences the oxide growth significantly [24], [40] (the water bridge). In fact, high concentration of oxyanions under the tip during the initial state of oxidation, as well as lateral oxyanions diffusion on the Si surface are important factors affecting the oxide growth [41]. The concentration of this oxyanions on the surface depends on the size of the water bridge and the electric field strength.

Fig. 7b indicates that the oxide thickness depends on the applied voltage for constant writing speed (0.6 µm/s) and RH% (68 RH%). As it can be seen, Au coated tip provides a thicker oxide layer for all applied voltages in comparison with the Cr/Pt coated tip, which is also applicable for different RH% (Fig. 7a). Since the oxide growth on Si surface was not significant below 5 V, this voltage can be considered as the threshold voltage for oxidation, when the writing speed is 0.6****µm/s.

**Figure 8:**
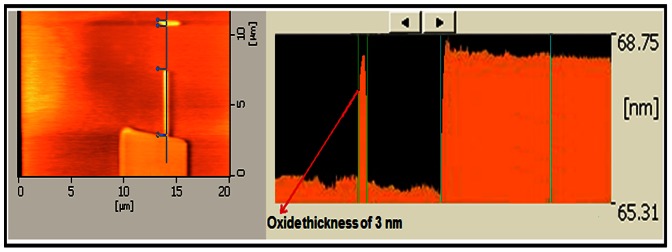
AFM images of the LAO patterned sample with the optimized parameter (Cr/Pt tip).

Humidity and temperature drastically affect the device structure during the patterning process. Normally to get a good pattern, the range of the temperature variation must be ≤4°C and the range of humidity variation must be ≤4%. For both Cr/Pt and Au coated tips, the observation reveals that the best results were in the range of 22–26°C for the temperature and 65–68% for the RH%. Fig. 8 shows the AFM topography and profile image of the arbitrary sample patterned by AFM –LAO, when the optimized parameters for Cr/Pt AFM tip (Table 3) are applied. The patterned structure is well-shaped and the thickness of 3 nm is an acceptable range for the oxide layer to act as a mask.

It is important to mention that applying high voltage may provide a thicker oxidation on the Si surface, but the pattern will be wider with lower uniformity. This can be improper for the purpose of nanodevice fabrication in order to extract the narrower nanowire after the etching process. Furthermore, for a high applied voltage in a high RH%, the shadow effect around the pattern will be inevitable.

### Shadow Effect

**Figure 9:**
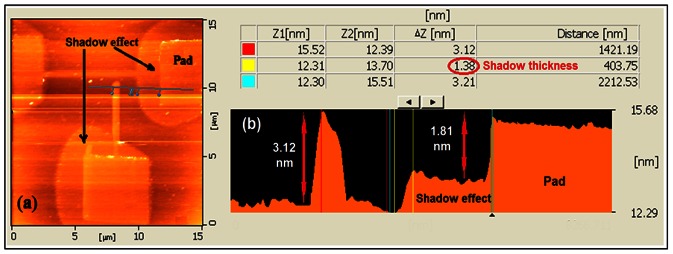
a) AFM image of the pattern after LAO in high RH% with shadow effect, b) profile view of the same sample showing the oxide and shadow thickness.

In the LAO process, the diffusion rate is proportional to the electric field strength. Larger applied voltage induces a larger electric field which causes a stronger interaction between the tip and the surface [41]. At high RH%, the electrochemical interaction between the tip and the surface becomes much stronger which prevents the effective oxidation at the center of the growing oxide. In this condition, the current expands horizontally through the surface layer, leading to the creation of shadows around the oxide pattern. According to our observation, when RH% was increased beyond 70%, the rate of increasing oxide thickness was reduced, and the shadow effect was observed. This supports the previous studies on AFM-LAO about the influence of room humidity in oxide growth on the Si surface, when the other parameters are constant [24], [41]. Fig. 9a presents the AFM topography image of the pattern with shadow effect around the square pads prepared by the LAO technique at high RH%. The AFM profile image for the same structure (Fig. 9b) presents the oxide thickness comparison for the pattern and shadow area around it. It shows that for the thickness of 3.12 nm for the oxide thickness, the shadow thickness is 1.38 nm around the oxide pattern.

### KOH Etching Process

Different research groups reported different wet and dry etching materials and processes. For instance, Hydrazine is one of the etchants with the advantage of negligible degradation during deep etching [10]; however, extreme caution is needed since it is highly toxic and potentially explosive. Dry plasma etching method, e.g. RIE method, is also employed. It allows obtaining a great variety of etching profiles ranging from isotropic etch to vertical sidewalls. However, the selectivity between the oxide mask and the silicon is generally poorer for liquid etchants [42].

The properties of non-annealed oxide created by AFM-LAO are different from thermally grown oxide, with a lower density and higher dielectric constant of 5.2 vs 3.9 [43]. Non-annealed oxide has even a greater H_2_O content by weight [44] in comparison with the thermal case. According to the low quality of the grown oxide by LAO, after the etching procedure, the selectivity of the agent is a critical parameter to achieve a high aspect ratio for nanostructures.

KOH saturated with IPA is a well-known etchant in the anisotropic chemical wet etching technique, which can used for the etching of nanostructures fabricated by the LAO technique. Several research teams have elaborately investigated the mechanism of the KOH anisotropic etching [45], [46], [47].

In order to extract the final structure after oxidation, two steps of etching process were conducted. KOH wet etching is a very significant part in the fabrication of devices. Some difficulties such as ill-etched, over etching or even contamination are hardly avoidable during the wet etching process. Accordingly, accuracy and precaution are essential to be given. Etching time (immersing time of a substrate in an etchant bath) depends on the thickness of the Si layer on top of the SOI wafer, which is desired to be removed. Considering the rate of the Si removal for KOH etching as 0.28 µm/min [48], the etching time of 20–22 s was selected to remove 100 nm top Si layer.

**Figure 10:**
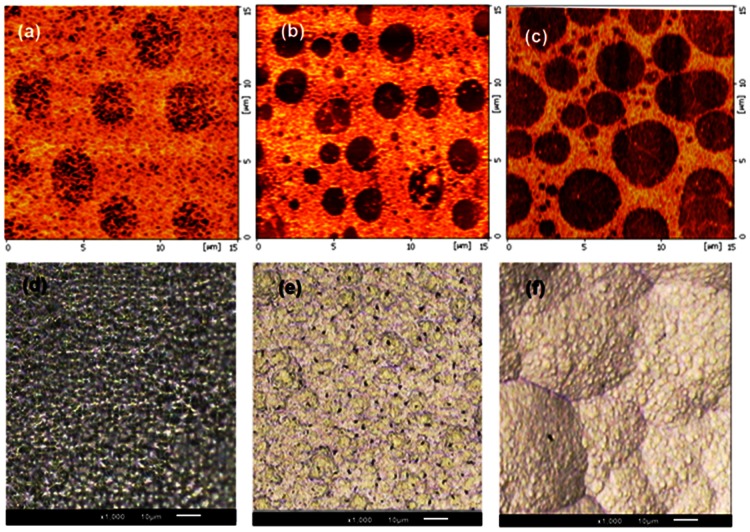
AFM images of the Si surface etched with KOH: (a) 10%wt (b) 20%wt (c) 30%wt, and SEM images of Si surface etched with 30%wt. **KOH +10%vol IPA at : d) 50°C (e) 60°C, (f) 80°C.**

Fig. 10(a–c) show AFM images of the etched substrates for different KOH percentage in solution (%wt) from 10% up to 30%. The solution was heated up to 63°C, stirred at 600 rpm. The substrate was immersed for 20–22 seconds and rinsed by DIW for a few seconds after being removed from the etching solution. It can be seen that the surface roughness is changed as the concentration of KOH increases up to 30%. At low KOH concentrations, the surface is rougher and the formation of insoluble precipitates can be observed. Roughness reduction of the Si surface, due to concentration increase, was reported in previous works [49], [50]. Similar observations were also reported in previous works [51], [52]. However, concentration is not the only main factor that determines the surface roughness; temperature and etching time are also contributing in the final roughness of the Si surface. In reported cases, the etching temperature was normally taken between 55°C−70°C to reach the acceptable edge cutting for patterned nanostructure.

It is approved that an admixture of isopropyl alcohol (IPA) in KOH etchant can improve the smoothness of the Si surface after etching [53]. Zubel*et al.* [54] reported the effect of the IPA concentration variation (2–12%) in the KOH etchant to enhance the smoothness of the surface. The impact of the IPA admixture in KOH etching was elaborately investigated in reported cases [45], [54]. In this work, IPA (60.10 g/mol supplied by Merck) was used as the initiator to improve surface roughness and control the etch rate [52]. It is important to mention that using IPA addition may slightly reduce the etching rate, but it definitely provides a smooth Si (100) surface in the all range of the examined concentration of KOH, specially for a high KOH ratio [55]. By controlling the temperature, in high KOH concentration with IPA addition, the etching rate can even be increased compare to the pure KOH [47]. Because of the low solubility of IPA in the KOH solution, there can be some limitation in analyzing the effect of IPA concentration on the smoothness of the etched surface.

Taking into account the natural evaporation of IPA during the etching process and its boiling point (80.37°C), the temperature during the etching process must be kept around 60°C to 80°C [45], [54], when the etching time is adjustable in accordance with the certain purpose of study. The effect of 30%wt. KOH+10% vol. IPA on quality of the etching surface for different temperatures was examined in constant etching time. SEM images of the etching effect on Si surface for different temperature (50°C to 80°C) are shown in Fig. 10 (d–f). The comparison shows smoother morphology at a lower temperature, whereby physical texture is much smaller compare to a higher temperature.

In the KOH etching process, the etching rate strongly depends on the water concentration [26]. Since water content of the solution extremely decreases at a higher level of the KOH concentration, then adding IPA can liberate water particles close to the Si surface to take part in the etching process. Meanwhile, an adsorption of IPA interrupts the access of OH**^−^** ions across the surface and reduces the oxidation rate, providing equilibrium in diffusion and oxidation of reaction products, which leads to more surface smoothness [46]. On the other hand, the decrease of the reaction rate, caused by IPA absorption, may lead to obtain the uniform etching on the Si surface.

During the KOH etching process, hydrogen bubbles are produced due to the reaction between the Si and the hydrogen ion. These bubbles increase the surface roughness [56] and act as surface masks against the reaction sites at the surface [54], [57] (“pseudo-mask” phenomenon [58]). The number and size of the hillocks during the Si etching are related to the hydrogen bubbles as well. The number of bubbles is directly proportional to the temperature, and the size of bubbles is reversely related to the etchant concentration. According to Yun [45], at temperatures below 70°C, the etch rate and concentration of KOH has the lowest influence on each other,. However, the faster etch rate can be achieved by using a high-temperature (>70°C) reaction. On the other hand, the sharpness of the etched structure is essential for nanodevices. The best sharpness after etching is achievable by keeping the temperature below 70°C and optimizing the KOH concentration. By increasing the concentration, it was observed that the density of bubbles increased but the size of the bubbles decreased. The same effect was already reported in previous studies [45]. In the range of our study, due to the low thickness of the Si layer, a high temperature may have a destructive effect on fabricated nanostructure.

During the fabrication of JLT devices, the effect of different KOH concentrations, mixed with 10%vol IPA, on the surface roughness of the devices was examined [59]. These results (not shown here) revealed that the surface roughness of the nanostrcture etched by 20%wt. KOH +10%vol IPA is higher than the one etched in 30%wt or 40%wt. KOH +10%vol. IPA. Considering the etchant solution (30%wt KOH +10%vol. IPA) for the best smoothness and sharpness, compare to the other examined etchant concentrations, the optimized range of etching temperature was extracted at 63–65°C. In this condition, compare to the case with 10%wt KOH +10%vol. IPA, the size of the bubbles is expected to be smaller. The small hydrogen bubbles leave the surface quickly, and the etching can be conducted much more efficiently by improving the sharpness of the structure. At 40%wt. KOH +10% IPA, produced hydrogen bubbles are expected to be smaller and the structure is anticipated to be sharper. However, the result was different and at this range, the sharpness and the smoothness were less than the case with 30%wt KOH. The reason can be explained by a faster Si etching at a high KOH concentration, where it can induce over etching on such structure with lower sharpness, in the range of applied temperature.

**Figure 11:**
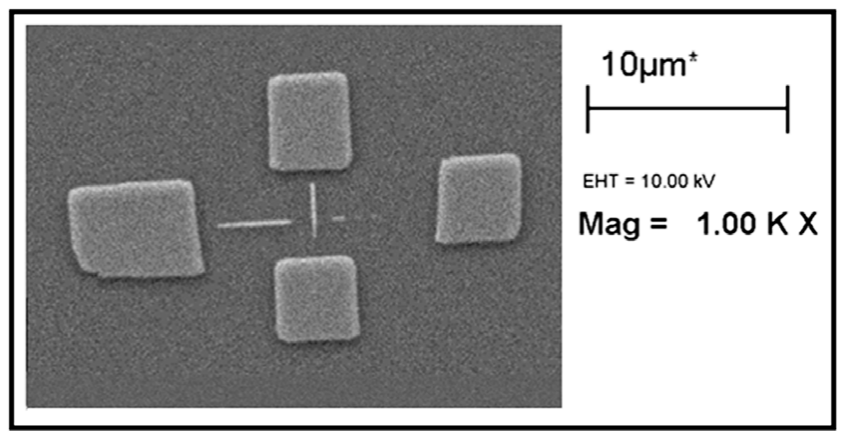
SEM image of the structure after etching with 30%wt. **KOH +10%vol. IPA at 63°C for 35 s.**

To investigate the role of the immersing time in the etching procedure for obtaining the optimum result, different etching times were examined. Fig. 11 shows the SEM image of the oxide pattern after etching. In this experiment, the sample was etched by 30%wt. KOH +10%vol. IPA solution at 63°C immersed for 35 seconds. Despite of the acceptable quality of the pads’ shape, the results revealed that most parts of the nanowire were gone. Long immersing time consequently destroyed the structure of the nanowire. The same effect is reported in [45], [60].

**Figure 12:**
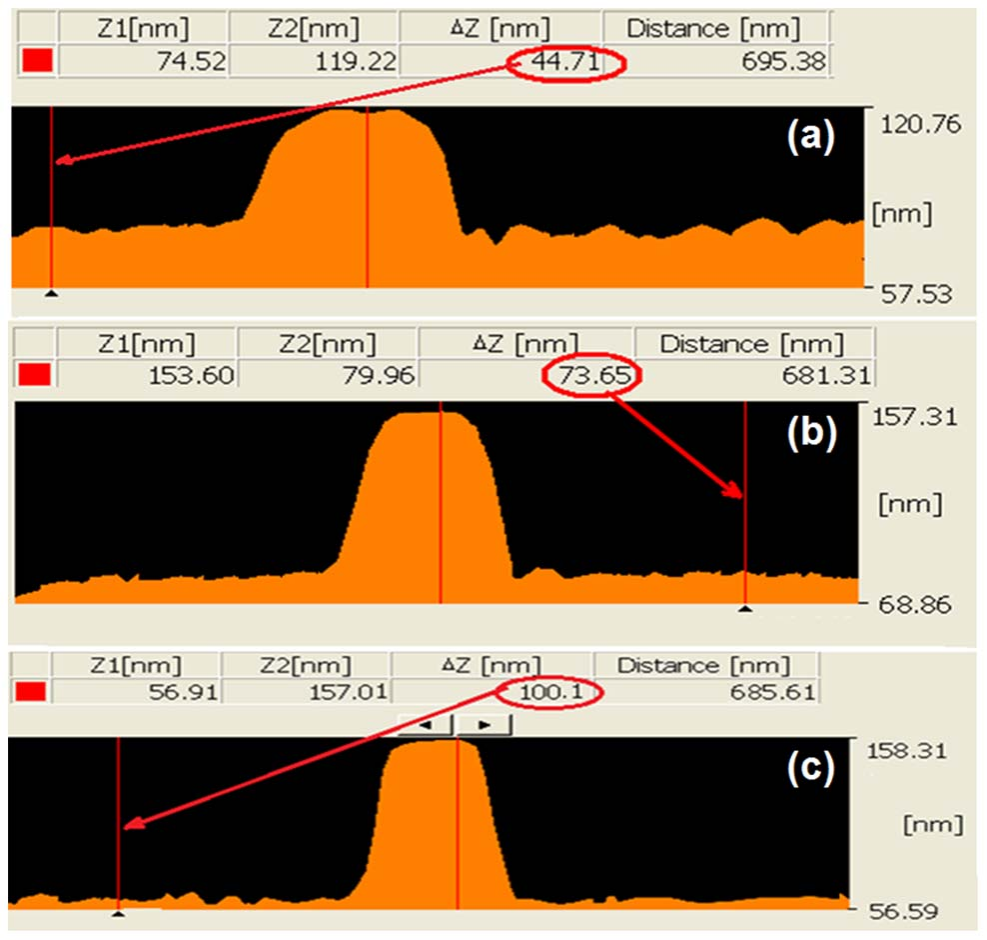
AFM profile images of the structure after etching for different immersing time, a) 8 s, b) 15 s, (c) 22 s (the solution of 30%wt. **KOH +10% IPA at 63**°**C).**

For shorter immersing time, the etch rate, as well as etched shape, can be different. For the solution of 30%wt. KOH +10% IPA at 63°C (optimized parameters), AFM profile images of the nanowire after etching for three different etching times are shown in Fig. 12. As it can be seen, for the shorter time of 8 s (a) and 15 s (b) compare to the longer time 22 s (c), the structures are under-etched, and the width of the nanowire are wider. This phenomenon can be related to different etch rates for the different crystallography planes. In anisotropic etching for Si (100), the etch pit is in a pyramid shape and the walls are flat and angled [45], [53]. For KOH etching, the etch rate for high index planes (e.g. (411), (311) or (211)) grows faster than the low index plane, like (110) or (111) [11], [53]. At shorter immersing time, high index planes of the Si surface would be more etched than lower index planes, and then the pit walls will be more angled. As it is expected, the etch depth for the shorter etching time, 44.71 nm for 8 s (Fig. 12a) and 73.65 nm for 15 s (Fig. 12b), are smaller than the etch depth (100.1 nm) after 22 s etching time (Fig. 12c). After 22 s of etching, the whole top Si layer (100 nm) was properly removed. Moreover, the addition of IPA to KOH solution can even more significantly reduce the etching rates of some high indexed crystal planes than the (1 0 0) plane [53].

### Optimum Condition for KOH Etching

**Figure 13:**
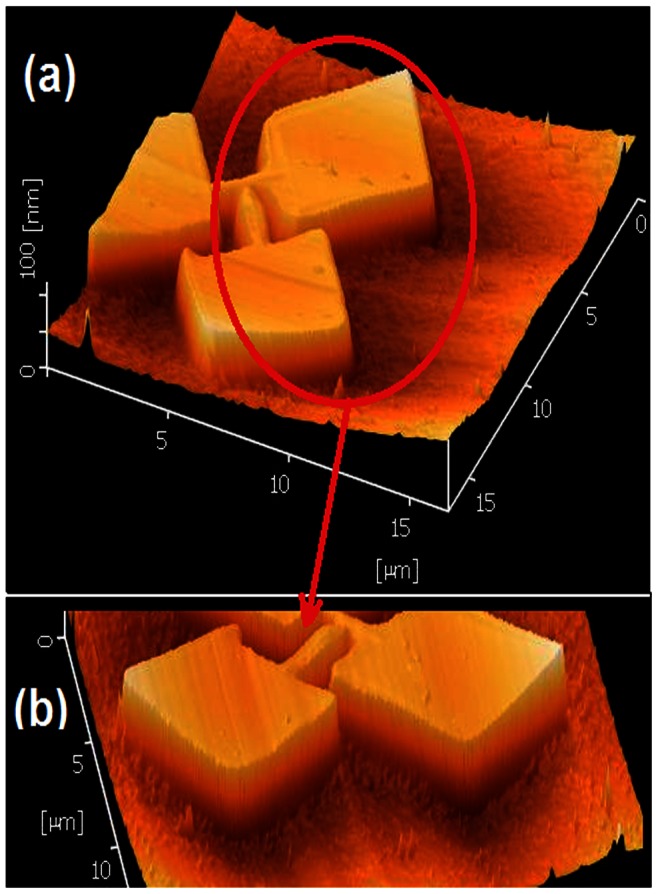
a) and b) AFM images of the structure after the optimized etching process.

In this study, previous works for obtaining the optimum condition of KOH etching were considered and adopted [56], [57], [60], [61]. The best condition according to the fabrication environment and other parameters is the solution of 30%wt. KOH with 10%vol. IPA for wet etching at 63°C, 22 seconds of immersing time, stirred at 600rpm. Stirring the solution is to ensure the uniformity of the etching process. The oxide removal was performed with diluted hydrofluoric acid (H_2_O/HF 100∶1) for the time period of 16 s to 18 s. Fig. 13(a–c) show AFM image and profile of the nanostructure for an arbitrary sample after the optimized etching procedure.

### Other Parameters and Issues

**Figure 14:**
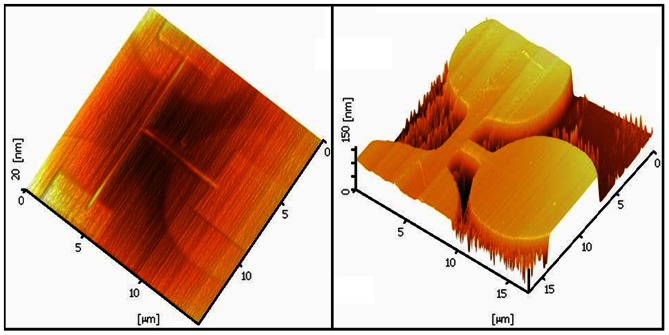
Shadow effect in the fabrication process on the SOI sample (a) after LAO, (b) after etching for the same sample.

In the fabrication process, in addition to the major factors (mentioned in previous sections), some minor factors may be able to make significant effects in the details. For instance, the size and geometry of the tip can have different impacts on the LAO process. Some other issues can be mentioned as the order of oxidation, hidrophobicity and dielectric constant of the Si material, doping concentration or even chemical composition of the atmosphere [62], [63]. The shadow effect can significantly affect the nanostructure after etching process. Fig. 14a demonstrates the structure with shadow effect after LAO. For the same sample after the etching process, the deformation of the whole structure can be seen in Fig. 14b, where the square shape turned into the round shape.

### Conclusions

In this paper, the impacts of different parameters in the fabrication of nanostructure on SOI by the improved AFM nanolithography were investigated. Sample preparation and cleaning process were modified from the RCA standard method. The impact of AFM-LAO parameters, such as the applied voltage on the tip, the relative humidity, the writing speed or the tip materials were studied to achieve the best structure. The shadow effect after the LAO process, due to the high relative humidity, was considered in this study. Two wet etching processes were implemented to extract the structure after the LAO process. The impact of the KOH concentration and KOH+IPA admixture for different temperatures on the SOI wafer were investigated. For different KOH concentrations, the effect of etching on the Si surface was also examined. The effect of the immersing time in the etching process was studied as well. The extracted optimized parameters for the etching process were 30%wt. KOH with 10%vol. IPA at 63°C, with the immersing time of 22 seconds and 600rpm stirring rate.
